# End Binding Proteins Are Obligatory Dimers

**DOI:** 10.1371/journal.pone.0074448

**Published:** 2013-09-06

**Authors:** Indrani Sen, Dmitry Veprintsev, Anna Akhmanova, Michel O. Steinmetz

**Affiliations:** 1 Laboratory of Biomolecular Research, Paul Scherrer Institut, Villigen PSI, Switzerland; 2 Cell Biology, Faculty of Science, Utrecht University, Utrecht, The Netherlands; University of Vienna, Max F. Perutz Laboratories, Austria

## Abstract

End binding (EB) proteins are responsible for the recruitment of an array of microtubule plus-end tracking proteins (+TIPs) to growing microtubules ends. EBs encompass an N-terminal calponin homology domain that confers microtubule tip tracking activity to the protein. The C-terminal domain of EBs contains a coiled coil that mediates the parallel dimerization of EB monomers. This part of the protein is also responsible for partner binding. While dimerization is not essential for microtubule tip tracking by EBs it is a prerequisite for +TIP partner binding. The concentration of EBs in cells has been estimated to be in the range of hundreds of nanomoles. In contrast, in *in vitro* single molecule experiments EB concentrations of subnanomoles are employed. From a mechanistic point of view it is important to assess the oligomerization state of EBs at physiologically and experimentally relevant protein concentrations, in particular if the goal of a study is to model the behavior of EB-dependent dynamic +TIP networks. Here we have determined the stability of the EB1 and EB3 dimers using multi-angle light scattering and fluorescence analytical ultracentrifugation. We show that these EBs form stable dimers and do not dissociate even at very low nanomolar concentrations. The dimers remained stable at both room temperature as well as at the physiologically relevant temperature of 37°C. Together, our results reveal that EBs are obligatory dimers, a conclusion that has implications for the mechanistic understanding of these key proteins involved in the orchestration of dynamic protein networks at growing microtubule ends.

## Introduction

Microtubule plus-end tracking proteins (+TIPs) constitute a unique group of structurally and functionally diverse proteins that target the plus ends of growing microtubules [1]. +TIPs are involved in many microtubule-based processes, including cell division, cell migration and intracellular trafficking [2]. End Binding proteins (EBs) are a highly conserved family of +TIPs [3]. They autonomously track growing microtubule ends and are responsible for the recruitment of other +TIPs to this location [4], [5]. As such, EBs orchestrate dynamic +TIP networks at microtubule ends [1], [6].

EBs contain an N-terminal calponin homology (CH) domain, which is necessary and sufficient for microtubule tip binding [7], [8]. The C-terminal domain of the protein encompasses an α-helical coiled coil, which is followed by a four helix bundle encoded by the unique and highly conserved EB-homology (EBH) domain and a disordered tail. The coiled coil and the EBH domain are responsible for the parallel homo- and heterodimerization of EB monomers and for +TIP partner binding [9]–[11]. The dimeric configuration of the EBH domain creates a prominent hydrophobic cavity at the interface between EB monomers, which together with the C-terminal tail region is responsible for +TIP partner binding [5], [12].

Since dimerization of EB subunits is crucial for EB function it is important to assess their oligomerization state at physiologically relevant concentrations or in the concentration regime used in *in vitro* reconstitutions studies aimed at deciphering the molecular mechanisms of EBs. The concentration of EBs in cells has been estimated to be in the range of hundreds of nanomoles [13]–[16]. In contrast, single molecule reconstitution experiments are performed with subnanomolar concentrations of EBs [4], [17]. Depending on the dissociation constant of the EB dimer, oligomerization can either take place spontaneously in the cytoplasm or be induced at the growing microtubule end due to an increased local concentration mechanism. To discriminate between these two possible models, we performed biophysical experiments with full length EB1 and EB3 and with their C-terminal dimerization domains. Our results suggest that EBs are obligatory dimers that self-assemble in the cytoplasm.

## Results and Discussion

**Figure 1:**
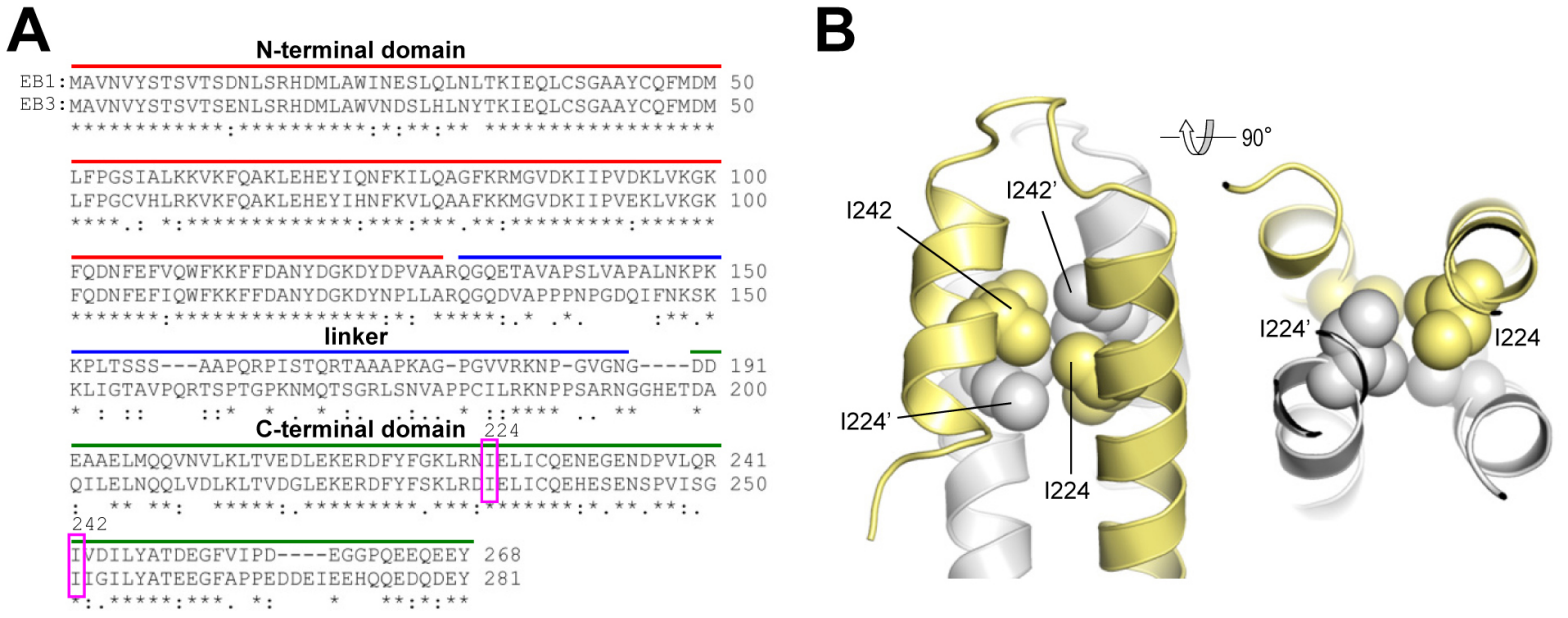
Sequence alignment of EBs and structure of the EBH domain. (**A**) Sequence alignment of full length human EB1 (accession number AAC09471) and EB3 (accession number BAA82958). The N-terminal domain, linker, coiled coil, EBH domain and tail region are indicated. The arrowhead highlights the isoleucine residue (Ile224 in EB1 and Ile233 in EB3), which was mutated to alanine to create EB monomers. (B) Two views 90° apart of the EBH domain of EB1 showing the core packing interactions of Ile224 and Ile242 (in sphere representation). Monomers A and B of the EBH domain are colored in grey and yellow, respectively, and are shown in cartoon representation. Residues of monomer B are indicated by a prime.

In this study we sought to assess the stability of the EB dimer. For this purpose we cloned GFP-tagged versions of human EB1 and EB3 (EB1-GFP and EB3-GFP) and of their C-terminal domains (EB1c-GFP and EB3c-GFP). We chose the ‘enhanced GFP’ version (EGFP; [18]) because this GFP variant is frequently used both in cellular and in *in vitro* reconstitution +TIP studies. The EGFP tag was fused to the C-termini of the proteins as the last approximately 20 amino acid residues of the EBs are disordered in solution [5] and thus not expected to interfere with dimerization. As a reference for the EB monomer, we made use of a residue substitution (I224A and I233A in EB1 and EB3, respectively; Figure 1A) that is known to abrogate EB dimer formation [9], [11] most likely by destabilizing the hydrophobic core of the EBH domain (Figure 1B). Bacterially expressed and affinity purified recombinant proteins were subsequently analyzed by multi-angle light scattering and analytical ultracentrifugation.

### Multi-angle Light Scattering

**Figure 2:**
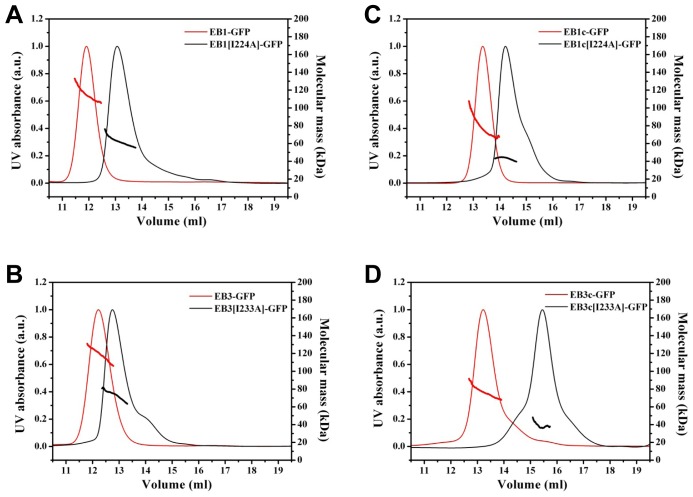
Multi-angle light scattering experiments of wild type and mutant EB proteins. Multi-angle light scattering experiments of EB1-GFP and EB1[I224A] (A), EB1c-GFP and EB1c[I224A] (B), EB3-GFP and EB3[I233A] (C), and EB3c-GFP and EB3c[I233A] (D). Molecular mass determination (horizontal lines located below the maximum of each peak) yielded the values reported in Table 1. a.u., arbitrary units.

To test the capacity of our purified EB proteins to form oligomers we used size exclusion chromatography (Superdex 200 10/30 column) coupled to a multi-angle light scattering instrument. As shown in Figure 2, all eight proteins displayed elution profiles consistent with the presence of predominantly single molecular species. EB1-GFP and EB3-GFP eluted at a volume of 11.9 and 12.1 ml, respectively (Figure 2, A and B; Table 1). In contrast, the mutants EB1[I224A]-GFP and EB3[I233A]-GFP eluted later at 13.1 and 12.8 ml, respectively (Figure 2, A and B; Table 1). Qualitatively identical results were obtained with the C-terminal domain fragments: Elution volumes of 13.3, 13.2, 14.2 and 15.4 ml were obtained for EB1c-GFP, EB3c-GFP, EB1c[I224A]-GFP and EB3c[I233A]-GFP, respectively (Figure 2, C and D; Table 1). We noted some tailing to higher volumes in the elution profiles of the monomeric EB protein variants, which primarily arose from the presence of degradation products.

**Table 1:** Biophysical data.

Protein	Elution volume^1^, ml	MW_calculated_ 2, kDa	MW^3^, kDa	s^4^, S
EB1-GFP	11.9	59.1	115 (0.6%)	4.9±0.12
EB1[I224A]-GFP	13.1	59.0	62 (0.3%)	3.2±0.15
EB1c-GFP	13.3	38.3	77 (2%)	4.4±0.12
EB1c[I224A]-GFP	14.2	38.2	43 (0.7%)	2.8±0.08
EB3-GFP	12.1	61.0	119 (1%)	4.6±0.23
EB3[I233A]-GFP	12.8	61.0	73 (1%)	3.3±0.04
EB3c-GFP	13.2	38.7	76 (0.7%)	3.8±0.31
EB3c[I233A]-GFP	15.4	38.7	39 (11%)	2.6±0.08

1 Elution volume of peaks obtained on a Superdex 200 10/30 size exclusion chromatography column.

2 Molecular weights calculated from the amino acid sequence.

3Molecular weights determined by multi-angle light scattering. The error of each measurement is given in parenthesis.

4 Sedimentation coefficients and corresponding standard deviations determined by fluorescence sedimentation velocity analytical ultracentrifugation.

The multi-angle light scattering signals measured under the major size exclusion chromatography peaks were used to calculate the molecular masses of the different EB proteins. We obtained molecular masses of 115 and 119 kDa for EB1-GFP and EB3-GFP (Figure 2, A and B; Table 1), consistent with both full-length EBs forming dimers (calculated molecular masses for the monomers are: EB1-GFP = 59.1 kDa; EB3-GFP = 61.0 kDa). The EB1[I224A]-GFP and EB3[I233A]-GFP mutants yielded a molecular mass of 62 and 73 kDa, consistent with the presence of monomers (Figure 2, A and B; Table 1). A similar pattern was observed with the C-terminal domain fragments: Molecular masses of 77, 76, 43 and 39 kDa were obtained for EB1c-GFP, EB3c-GFP, EB1c[I224A]-GFP and EB3c[I233A]-GFP, respectively (calculated molecular masses for the monomers are: EB1c-GFP = 38.3 kDa; EB3c-GFP = 38.7 kDa; Figure 2, C and D; Table 1). Together, these results confirm that the C-terminal domain is responsible for the dimerization of EB monomers, as previously reported [9]–[11].

### Analytical Ultracentrifugation

The multi-angle light scattering experiments described above indicate that EBs form stable dimers at a concentration of ∼1 µM (estimated from the OD_280_ signal at the peak position of the size exclusion chromatography profile). To assess the oligomerization state of our proteins at lower concentrations we switched to fluorescence sedimentation velocity analytical ultracentrifugation, which allows measuring GFP-tagged proteins down to a concentration of about 1 nM.

**Figure 3:**
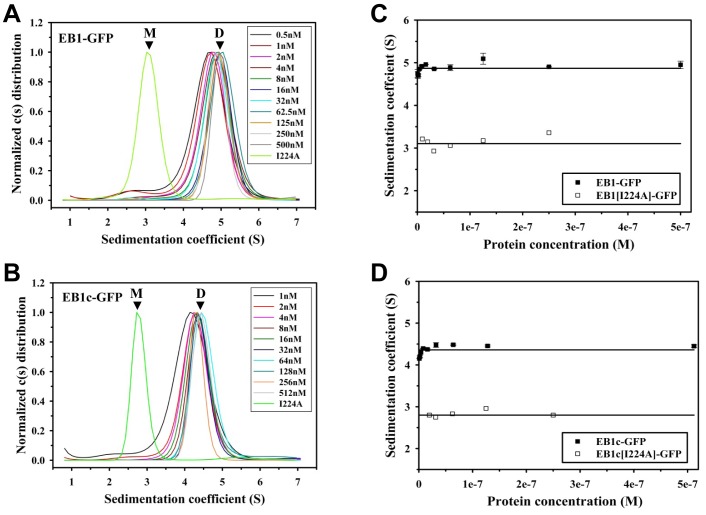
Sedimentation velocity experiments of GFP-tagged EB1 proteins. Sedimentation coefficient distribution profiles of EB1-GFP (A) and EB1c-GFP (B) at the different protein concentrations indicated in the corresponding legends. The I224A mutant versions of the proteins are also shown as a reference. Sedimentation coefficient positions of monomers (M) and dimers (D) are indicated. (**C**) and (**D**) Sedimentation coefficients (symbols) plotted against protein concentration for EB1-GFP and EB1[I224A]-GFP (C), and EB1c-GFP and EB1c[I224A]-GFP (D). The lines represent the linear fits to the data.

**Figure 4:**
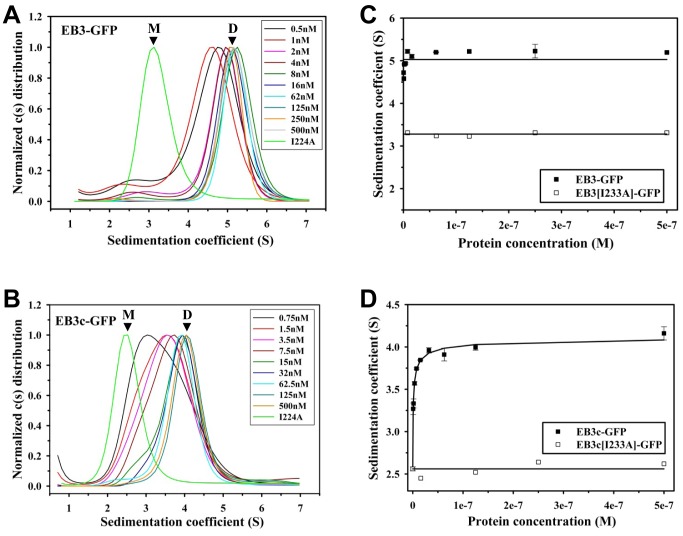
Sedimentation velocity experiments of GFP-tagged EB3 proteins. Sedimentation distribution profiles of EB3-GFP (A) and EB3c-GFP (B) at the different protein concentrations indicated in the corresponding legends. The I233A mutant versions of the proteins are also shown as a reference. Sedimentation coefficient positions of monomers (M) and dimers (D) are indicated. (**C**) and (**D**) Sedimentation coefficients (symbols) plotted against protein concentration for EB3-GFP and EB3[I224A]-GFP (C), and EB3c-GFP and EB3c[I224A]-GFP (D). The line in (C) represents the linear fit to the data. The curve in (D) represents the fit to the data assuming a monomer-dimer self-association model (K_d_ = 2 nM).

As shown in Figure 3 and 4, at a concentration of ∼500 nM (monomer equivalents) and 22°C, all eight proteins tested revealed a cumulative sedimentation coefficient distribution profile, c(s), suggestive of single molecular species. The mean sedimentation coefficients, s, obtained for EB1-GFP and EB3-GFP were 4.9 and 4.6 S, respectively (Figure 3A; Figure 4A; Table 1). Based on the results obtained by size exclusion chromatography and multi-angle light scattering (Figure 2; Table 1), we assigned these s values to EB dimers. The corresponding s values for the monomeric versions were determined by analyzing the mutant versions of EB1 and EB3. As shown in Figures 3A and 4A, mean s values of 3.2 and 3.3 S were obtained for EB1[I224A]-GFP and EB3[I233A]-GFP, respectively; these values were assigned to EB monomers. Qualitatively identical results were obtained with the C-terminal domain fragments: the mean s values of EB1c-GFP, EB3c-GFP, EB1c[I224A]-GFP and EB3c[I233A]-GFP were determined as 4.4, 3.8, 2.8 and 2.6 S, respectively (Figures 3B and 4B; Table 1).

Next we measured by fluorescence sedimentation velocity dilution series of our protein samples in a concentration range from ∼500 to ∼0.5 nM (monomer equivalents) and used the gradual shift of the observed s values from ∼5 to ∼3 S for full-length EB or from ∼4 to ∼2.7 S for C-terminal EB domains as a measure to monitor the dissociation of EB dimers (Figure 3 and 4). By plotting the s values against protein concentration we obtained a dissociation isotherm for each protein sample. As shown in Figure 3C and Figure 4C, EB1-GFP and EB3-GFP remained dimeric down to a concentration of 0.5 nM. Similarly, EB1c-GFP did not dissociate significantly into monomers in the same protein concentration range (Figure 3D). In contrast, significant amounts of EB3c-GFP monomers were detected when the concentration of the protein was below 15 nM (Figure 4D). The EB3c-GFP dissociation isotherm could be fitted to a monomer-dimer self-association model, which yielded an apparent dissociation constant, K_d_, of 2 nM. The lesser stability of EB3c compared to EB1c has been previously reported based on circular dichroism spectroscopy measurements [9]. Moreover, the result that full length EB3 is more stable than EB3c is consistent with small angle X-ray scattering data suggesting that all four EB elements, CH domain, linker sequence, coiled coil and EBH domain collectively contribute to the high stability of the full length EB dimer [19].

**Figure 5:**
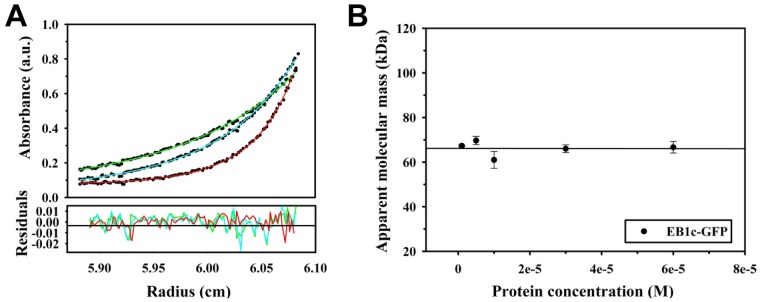
Sedimentation equilibrium data at 37°C for EB1c-GFP. (**A**) Sedimentation equilibrium profiles obtained with 1 µM EB1c-GFP at three different rotor speeds (14′000 rpm (green), 17′000 rpm (blue), 26′000 rpm (red). (**B**) Plot of protein concentration versus molecular mass. The symbols represent data points; the line the linear fit to the data.

To test whether the EBs are less stable at physiologically relevant temperatures, we performed absorption sedimentation equilibrium experiments at 37°C (note that it is technically not possible to perform fluorescence sedimentation velocity experiments above room temperature). We carried out experiments with the C-terminal domain of EB1 because we observed that the full length EB proteins were susceptible to degradation under the experimental conditions used, most likely because of the flexible nature of the ∼70-residue long linker region between the N- and C-terminal domains of EBs. As shown in Figure 5, EB1c-GFP remained dimeric over a concentration range from 1 to 60 µM (monomer equivalents; note that below 1 µM protein concentration we were not able to obtain accurate data by absorption sedimentation equilibrium). This result indicates that also at physiological temperatures EBs form stable dimers.

### Influence of the GFP Tag on the Stability of EB1 Dimers

**Figure 6:**
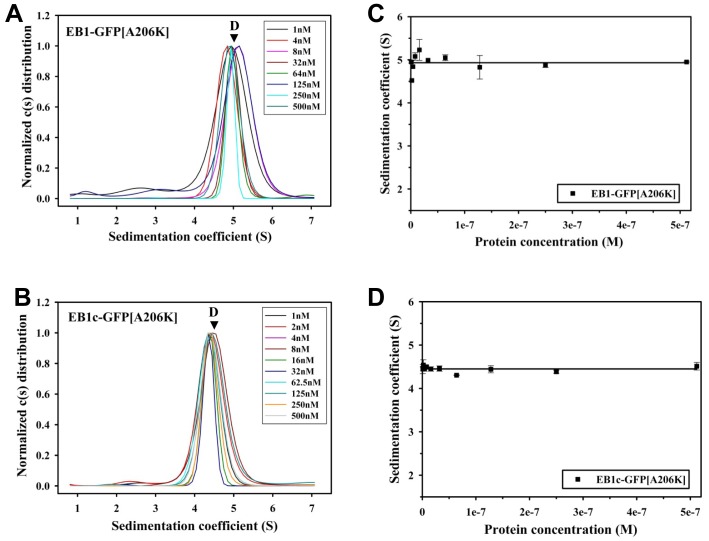
Sedimentation velocity experiments of GFP[A206K]-tagged EB1 proteins. Sedimentation coefficient distribution profiles of EB1-GFP[A206K] (A) and EB1c-GFP[A206K] (B) at the different protein concentrations indicated in the corresponding legends. Sedimentation coefficient positions of dimers (D) are indicated. (**C**) and (**D**) Sedimentation coefficients (symbols) plotted against protein concentration for EB1-GFP[A206K] (C) and EB1c-GFP[A206K] (D). The lines represent the linear fits to the data.

It is well established that GFP has a weak tendency to form dimers in solution (K_d_ = 0.11 mM; [20]). To test whether this dimerization activity has a significant effect on the stability of our GFP-tagged EB variants, we mutated Ala206 of the EGFP tag to lysine in EB1-GFP and EB1c-GFP (EB1-GFP[A206K] and EB1c-GFP[A206K]); this residue substitution is known to abrogate dimerization of GFP [20]. Sedimentation velocity experiments showed that the mutation did not have any significant effect on the stability of both the EB1-GFP and EB1c-GFP dimers (Figure 6). This finding suggests that C-terminal tagging of EBs with wild-type GFP does not significantly introduce additional, non-native interactions in EB-GFP fusion constructs.

### Conclusions

Our multi-angle light scattering and analytical ultracentrifugation data suggest that EBs form dimers and remain dimeric at low nanomolar (0.5 nM) protein concentrations. Single molecule *in vitro* reconstitution assays use subnanomolar concentrations of EBs [4], [17]. Our analysis suggests that in such a low concentration regime EBs would still form predominantly dimers. However, since the concentration of EBs in cells has been estimated to be in the range of hundreds of nanomoles [13]–[16] our results define EBs as obligatory dimers that self-assemble in the cytoplasm and track growing microtubule plus ends as dimers. A possible consequence of this result is that +TIPs may already bind to EB dimers in the cytoplasm and localize as +TIP-EB complexes to growing microtubule ends. Alternatively, +TIPs get primarily recruited by EB dimers already present at microtubule tips. These considerations should have implications for the computational modeling of dynamic +TIP networks and microtubule plus-end tracking processes.

## Experimental Procedures

### Cloning, Expression and Protein Purification

C-terminally EGFP-His-tagged human EB1, EB1[I224A], EB3, EB3[I233A], EB1c (Asp191-Tyr268), EB1c[I224A], EB3c (Ala200-Tyr281) and EB3c[I233A] were cloned into the pET28a cloning vector (Invitrogen). The A206K GFP mutation in EB1-GFP and EB1c-GFP was introduced by a PCR-based mutagenesis strategy.

Proteins were expressed in the *E. coli* strain BL21(DE3) (Invitrogen) in LB medium. Inoculated cultures were grown at 37°C until an OD_600_ of 0.8 was reached, induced with 1 mM IPTG, and incubated for 16 hours at 20°C. Proteins were purified by immobilized metal affinity chromatography using HisTrap™ HP Ni^2+^-Sepharose columns (GE Healthcare) at 4°C using standard protocols. Proteins were further processed by size exclusion chromatography in 20 mM Tris-HCl, pH 7.5, 300 mM NaCl, 5 mM beta-mercaptoethanol, concentrated to approximately 5 mg/ml, aliquoted, flash frozen in liquid N_2_, and stored at −80°C.

The homogeneity and purity of protein samples were assessed by Coomassie stained SDS-PAGE and mass spectrometry. The purity of the protein preparations right after purification was typically >90%. We noted that the monomeric EB versions were more prone to degradation over time compared to the dimeric, wild type proteins.

### Multi-angle Light Scattering

Standard multi-angle light scattering experiments were carried out on a miniDawn TriStar system connected in-line to an Optilab rEX refractometer (Wyatt Technology Corporation) coupled to a Superdex 200 10/30 (GE Healthcare) run on an HPLC system (Agilent 1100). 1 to 4 mg/ml samples were injected in a volume of 100 µl at a flow rate of 0.5 ml/min onto the column equilibrated with 20 mM Tris-HCl, pH 7.5, 300 mM NaCl, 2 mM DTT. Molecular weights and standard deviations were determined using the Astra software package version 5.3.4. (Wyatt Technology Corporation). All experiments were performed at room temperature. The molecular weight values reported in Table 1 were derived from single experiments.

### Analytical Ultracentrifugation

Samples for sedimentation velocity experiments were diluted in 20 mM Tris-HCl, pH 7.5, 300 mM NaCl supplemented with 0.2 mg/ml bovine serum albumin (BSA) and either with 2 mM DTT or 0.1 mM TCEP. Prior to loading the samples, cells were washed with 1 mg/ml BSA to avoid unspecific binding. Sedimentation velocity experiments were performed in an Optima XL-1 (Beckmann) analytical ultracentrifuge at 22°C using charcoal-filled epon double-sector velocity cells and sapphire glass windows. All the samples were measured in duplicates. A fluorescence detection system (Aviv Biomedicals) was used for all the velocity runs to monitor the GFP fluorescence signal at 488 nm. The partial specific volume as well as the solvent density and viscosity were calculated using SEDNTERP (http://bitcwiki.sr.unh.edu). Data analysis was performed using the software package SEDFIT [21]. All samples were incubated for 16 hours at 22°C prior to measurement. The mean sedimentation coefficient values and corresponding standard deviations reported in Table 1 were calculated from at least 5 individual experiments.

For sedimentation equilibrium experiments, protein dilutions were prepared as described above but without BSA in the buffer. Experiments were performed at 37°C in triplicates using the charcoal-filled epon six sector cells. Equilibrium was reached for the samples at the three speeds of 14′000, 17′000 and 26′000 rpm. Data analysis was performed using the Ultraspin software package (D. Veprintsev, http://www.mrc-lmb.cam.ac.uk/dbv/ultraspin2/).

An An-50 Ti rotor (Beckmann) was used for both sedimentation velocity and sedimentation equilibrium experiments. The stability of protein sample before and after an experiment was assessed by Coomassie stained SDS-PAGE.
